# A case report of three children with secondary hypertension caused by Liddle syndrome 

**DOI:** 10.5414/CNCS109972

**Published:** 2020-05-29

**Authors:** Zheyi Teoh, Siddharth Shah

**Affiliations:** Department of Pediatrics, University of Louisville, Louisville, KY, USA

**Keywords:** Liddle syndrome, secondary hypertension, monogenetic hypertension

## Abstract

Background: Liddle syndrome is a monogenetic cause of early-onset hypertension that is associated with hypokalemia and metabolic alkalosis that is inherited in an autosomal dominant fashion with variable penetrance. Case presentation: We present a case report of three children seen at a tertiary children’s hospital with varying severity of hypertension and electrolyte disturbances, who had genetic testing performed due to strong family history of hypertension. They were each subsequently found with the same genetic mutation of SCNN1B consistent with Liddle syndrome and started on epithelial sodium channel inhibitors with improvement in their blood pressure. Conclusion: Due to its variable penetrance, Liddle syndrome can have varying severity of blood pressure and electrolyte disturbances. Prompt recognition of Liddle syndrome is important to prevent cardiovascular complications from uncontrolled hypertension.

## Background 

Liddle syndrome was originally described in 1963 [1] as a case report of a family of siblings with early-onset hypertension, hypokalemia, and metabolic alkalosis. Although this appeared similar to primary hyperaldosteronism, these patients had suppressed renin and aldosterone levels, which did not improve despite a low salt diet challenge. They also did not demonstrate improvement to mineralocorticoid receptor antagonists but marked response to a direct epithelial sodium channel (ENaC) inhibitor, suggesting an intrinsic defect of these channels. A case study published 30 years later on a family member originally described by Liddle reaffirmed the hypothesis of an intrinsic renal defect, when that patient had resolution of symptoms following a cadaveric renal transplant [2]. 

Liddle syndrome is now better understood and is known to be inherited in an autosomal dominant manner with variable penetrance. The defect has been localized to various mutations of the β-, or γ-subunit of ENaC in the distal collecting tubules [3, 4, 5]. This causes constitutively active channels which increases sodium reabsorption and, in most cases, potassium and hydrogen ion wasting, simulating the effects of hyperaldosteronism [6]. 

Currently, Liddle syndrome is suspected in hypertensive young patients, especially those with a family history of early-onset hypertension and hypokalemia (although neither features are present in mild or atypical cases) [5, 7]. There appears to be no predilection for a particular gender or race/ethnicity, and the syndrome has been described worldwide [5]. Other supporting laboratory findings include metabolic alkalosis and low plasma renin and aldosterone concentrations with diagnosis confirmed by genetic sequencing of the sodium channel epithelial 1 β/γ subunit (*SCNN1B/SCNN1G*) [5]. Treatment mainly involves use of a direct ENaC inhibitor such as amiloride or triamterene to control blood pressure and mitigate downstream cardiovascular effects of hypertension. 

Currently, few case reports exist describing different patient characteristics and presentation of Liddle syndrome. We describe three cases of Liddle syndrome who had varying presentations despite the same genetic mutation. 

## Case 1 

A 9-year-old female presented to the emergency department with several days of dizziness, confusion, chest pain, blurry vision, and headaches. On initial evaluation, she was noted to be hypertensive, confirmed on repeat manual measurements, which was as high as 178/118 (> 99^th^ percentile). Initial investigations included a comprehensive medical panel which showed mildly elevated sodium of 149 mmol/L and hypokalemia (3.5 mmol/L) with a normal bicarbonate (27 mmol/L), blood urea nitrogen (9 mg/dL), and creatinine (0.6 mg/dL). 

**Figure 1:**
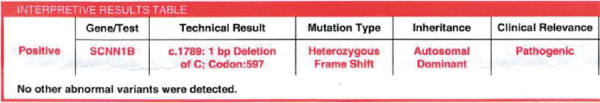
Genetic results for case 1.

Due to severity of hypertension, further workup included a normal EKG and echocardiogram without evidence of left ventricular hypertrophy, normal head CT scan, as well as a normal renal ultrasound and MAG-3 renal scan without evidence of renal artery stenosis. Thyroid function, metanephrines, and free cortisol were all within normal limits, but renin was low at < 0.1 ng/mL/h and aldosterone low at < 3.0 ng/dL. Given the findings of decreased renin and aldosterone, as well as a significant family history of early-onset hypertension in the mother, an underlying genetic disorder was suspected. A monogenic hypertension genetic panel was sent which returned as shown in Figure 1. 

Although she initially remained normotensive without treatment, she eventually required initiation of amiloride and low salt diet with subsequent maintenance of blood pressure in subsequent office visits. 

## Case 2 

**Figure 2:**
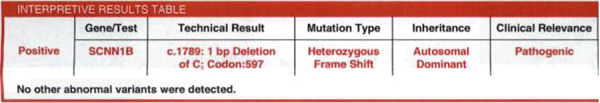
Genetic results for case 2.

An 8-year-old male presented to the hospital for dizziness, headache, and intermittent chest pain and was found to be persistently hypertensive at 160/100 (> 99^th^ percentile). Initial comprehensive metabolic panel was notable for mild hypernatremia (147 mmol/L), and hypokalemia (3.1 mmol/L) with a normal bicarbonate (23 mmol/L), blood urea nitrogen (9 mg/dL), and creatinine (0.5 mg/dL). An EKG demonstrated evidence of left ventricular hypertrophy which was later confirmed by an echocardiogram. He was admitted to hospital for further workup, and Pediatric Nephrology was consulted. A workup including thyroid function, vanillylmandelic acid, cortisol, and renal ultrasound with Dopplers were normal, but renin and aldosterone levels were both low at < 0.1 ng/mL/h and < 1.6 (ng/dL), respectively. Review of family history was significant for hypertension in his father, paternal grandfather, and half-brother (from his father’s side) including early-onset hypertension in his father at 13 years old and half-brother at 9 years old. Given the significant family history and hypokalemia, Liddle syndrome was suspected, and genetic studies were sent which was positive as shown in Figure 2. 

He has since been managed with amiloride and amlodipine with improved control of his blood pressure. 

## Case 3 

**Figure 3:**
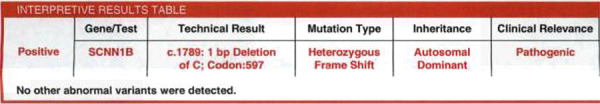
Genetic results for case 3.

A 15-year-old male who was initially diagnosed with hypertension as a 9-year-old represents to Pediatric Nephrology clinic after being lost to follow-up and after his half-brother (case 2) was diagnosed with Liddle syndrome. As a 9-year-old, his initial investigation included a renal functional panel that showed mild hypokalemia (3.6 mmol/L) but normal bicarbonate (26 mmol/L), blood urea nitrogen (13 mg/dL), and creatinine (0.6 mg/dL). A renal ultrasound and echocardiogram was normal without evidence of left ventricular hypertrophy. Renin and aldosterone levels at that time were decreased (< 0.1 ng/mL/h and < 1.6 ng/dL, respectively). He was initially managed with amlodipine and amiloride, but due to non-compliance, his blood pressure remained persistently elevated at his primary care doctor’s. On return to Pediatric Nephrology clinic, he had genetic testing performed which confirmed the same genetic mutation as his half-brother (Figure 3). 

Given the confirmation of Liddle syndrome, he was restarted on amiloride with an emphasis on compliance. [Table Table1]

## Discussion 

### Workup of pediatric hypertension 

Unlike their adult counterparts, pediatric patients with hypertension are more likely to have secondary causes of hypertension [8]. An initial workup for pediatric hypertension targets the top causes of secondary hypertension such as renal parenchymal or vascular disease, cardiac disease including coarctation, and endocrine disorders such as pheochromocytoma, Cushing’s syndrome, and hyperthyroidism [8, 9, 10]. 

The presence of severe hypertension, electrolyte disturbances, and a strong family history of hypertension should further raise suspicion for a monogenic cause of hypertension. Monogenic hypertension is a group of disorders (including but not limited to Liddle syndrome, Gordon’s syndrome, and familial hyperaldosteronism) characterized by single genetic mutations that are inherited in a Mendelian fashion [5, 10]. Because these disorders uniformly have suppressed renin [10], serum renin should be performed when these disorders are suspected. Serum aldosterone should also be performed concurrently, as they are suppressed in certain monogenic disorders (such as Liddle syndrome and apparent mineralocorticoid excess syndrome) and elevated in others [5, 10]. Further genetic studies can help identify specific mutations and guide anti-hypertensive therapy since these disorders generally do not respond to conventional treatments. 

### Characteristics of Liddle syndrome 

Because Liddle syndrome has variable penetrance, presentation can vary across age ranges and can include asymptomatic hypertension to hypertensive crisis. However, the presence of severe hypertension in the setting of electrolyte disturbances and a positive family history should clue providers into the possibility of Liddle syndrome. 

Our case report demonstrates the varying presentations of Liddle syndrome caused by the same genetic mutation (*SCN1BB* frameshift mutation) in the same geographic location. Presentation ranged from asymptomatic hypertension to hypertensive crisis and each patient had varying degrees of hypokalemia and none had metabolic alkalosis. However, markedly suppressed levels of renin and aldosterone were uniform, and all three patients responded well to amiloride, an agent that specifically targets the constitutively active ENAC channels. Untreated, Liddle syndrome can lead to significant cardiovascular mortality and morbidity, and prompt recognition is paramount. 

## Conclusion 

The presence of early-onset hypertension accompanied by a strong family history and electrolyte disturbances should raise concern for monogenetic causes of hypertension such as Liddle syndrome. Liddle syndrome can have varying presentations including different severities of blood pressure and electrolyte disturbances. 

## Funding 

The authors are not currently in receipt of any research funding relating to the research presented in this article. 

## Conflict of interest 

ZT and SS declare that they have no conflict of interest to declare. 

**Table 1. Table1:** Table 1. Summary of case characteristics.

Factor	Case 1	Case 2	Case 3
Age at presentation	9	8	9
Presenting symptom	Hypertensive urgency	Hypertensive urgency	Asymptomatic hypertension
Family history	Mother with early-onset hypertension	• Paternal half-brother with Liddle’s syndrome • Father and paternal grandfather with early-onset hypertension	
Initial plasma potassium (mmol/L)	3.5	3.1	3.6
Initial plasma HCO_3_ (mmol/L)	27	23	26
Serum renin (ng/mL/h)	< 0.1	< 0.1	< 0.1
Serum Aldosterone (ng/dL)	< 3.0	< 1.6	< 1.6
Genetic findings	*SCNN1B* frameshift mutation
